# Improving wheat seedling quality through deep ploughing and soil compaction at sowing in lime concretion black soil

**DOI:** 10.1371/journal.pone.0288459

**Published:** 2023-07-11

**Authors:** Xuejun Cui, Zhiwei Wang, Tengfei Zhuang, Jianqiang Sun, Youhong Song

**Affiliations:** 1 School of Agronomy, Anhui Agricultural University, Hefei, Anhui Province, China; 2 Lu’an Academy of Agricultural Sciences, Lu’an, Anhui Province, China; 3 Development Bureau of Crop Farming of Mengcheng County, Mengcheng, Anhui Province, China; Directorate of Rapeseed-Mustard Research, INDIA

## Abstract

The straw incorporation in lime concretion black soil compromises the emergence and quality of winter wheat seedlings in Huaibei Plain, China, lowering the potential of wheat productivity. To overcome the disadvantage, a two-year field experiment was conducted in 2017–18 and 2018–19 to investigate the effects of different tillage modes on seedling emergence and subsequent seedling growth, and final grain yield (GY) in winter wheat. The modes are rotary tillage with compaction after sowing (RCT), rotary tillage after deep ploughing (PT) and rotary tillage after deep ploughing with compaction after sowing (PCT), with the traditional rotary tillage (RT) method as the control. Compared to RT, greater soil moisture content (SMC) at the seedling stage was observed in deep ploughing or compaction treatment, and the highest SMC was achieved in PCT; the time of reaching the maximum number of seedlings was 1 d sooner in RCT or PT, and 3 d in PCT; the seedling number in RCT, PT and PCT was significantly increased by 32.6%, 34.5% and 61.5% respectively. The population size, shoot and root growth of winter wheat in ploughing mode was significantly enhanced than that of rotary treatment at the over-wintering stage; compared to no compaction after sowing, plant growth in compaction treatments was significantly promoted with greater plant population size and height of seedlings. At harvest, GY in RCT, PT and PCT was significantly improved by 5.87%, 10.8% and 16.4%, respectively, compared to RT and the highest GY was achieved in PCT by up to 8, 350.1 kg ha^-1^ due to the increased spike number. In conclusion, the seedling quality in the straw incorporation practice was improved through rotary after deep ploughing and compaction after sowing for lime concretion black soil in Huaibei Plain, China or a similar soil type.

## 1. Introduction

Wheat (*Triticum aestivum* L.) belongs to the genus *Triticum* of the family Gramineae, which is the first food crop cultivated by human beings [1]. In China, wheat is one of most important staple food crops, critical for national food security [2]. Huaibei Plain in Anhui Province is located at the south of Huang-Huai-Hai Plain, where it is an important crop production region in China. An annual rotation system of winter wheat-summer maize is mainly adopted in this area [3, 4]. Shajiang black soil namely lime concretion black soil, has the characteristics of high soil compactness, large farming obstacles, low soil organic matter and poor soil fertility [5, 6]. Huaibei Plain accounts for more than 60% of national lime concretion black soil farmland area. This region belongs to the warm temperate climate area with an obvious alternation of dry and wet seasons [7]. Crop production in this area is mainly dependent on rainfall due to limited irrigation resources. Therefore, it is challenging to achieve a stable and high yield for winter wheat in Huaibei Plain, China or the similar areas.

Crop straw retention is beneficial for increase of soil organic matter and nutrients [8, 9], and improvement of soil physical and chemical properties [10, 11]. In general practice, maize residues are directly returned to the top soil layer within 10-15cm prior to winter wheat sowing in Huaibei Plain. Despite positive effects on soil structure and fertility, it is noted that straw retention may cause adverse effects on wheat cropping. For instance, the poor seedling quality is often caused by maize straw retention due to an increase in soil pores [5, 12]; in addition, the straw retention resulted in inconsistent sowing depth, which led to the unevenness of seedling emergence, more seedling deficiency, decreased quality of seedling emergence [13]. On the other hand, the continuous rotary tillage for many years resulted in shallower loose topsoil, and a plough layer underneath the top soil, which caused soil compactness and poor permeability in ventilation [14–16]. In brief, the conventional tillage practice with crop straw incorporation caused compromised seed germination, even seedling death due to shallow soil layers and huge amount of straw buried in the soil [17, 18]. However, it is the prerequisite to achieve stable and high crop yield by having produced robust seedlings, and the improvement in soil preparation and sowing quality are the key technical measures in fostering strong wheat seedlings prior to the winter season.

Studies showed that the utilisation of deep ploughing caused reduced compactness of deep soil, improved state of soil pores, and promoted growth of root system [16, 19–21]. Although deep ploughing may increase the input of production cost compared with conventional farming technologies, the economic benefit of deep ploughing for wheat production was significantly improved [22]. Despite that the unduly compacted soil limited water and nutrient availability for crop roots [23], the moderate soil compaction after sowing has some positive effects on winter wheat growth [24]. Soil compaction after winter wheat sowing become an important cultivation practice, widely applied in Huang-Huai-Hai Plain in recent years. The moderate soil compaction could increase the root-soil contact, allowing the crop root to extract adequate resources [25]. In some soil types, especially coarse-textured soils, soil compaction could reduce percolation losses, and increase soil water storage, thus enhancing crop yield [26, 27]. What’s more, the moderate soil compaction significantly improved grain yield by increasing the number of effective spikes at maturity through raising the number of tillers of winter wheat seedling, especially the large tillers before winter [28]. In general, the adequate soil moisture at seedling stage can ensure the growth of seedlings and help to generate strong seedlings, laying a foundation for the high production of winter wheat. However, in the Huaibei Plain, the winter wheat is prone to drought at sowing or seedling stage. Therefore, it is critical to ensure the optimum soil moisture during the seedling stage by virtue of advanced cultivation strategies in Huaibei Plain.

The previous research of deep ploughing and compaction in wheat sowing is mainly focused on the effects of soil physical and chemical properties on grain yield, however wheat seedling quality under different tillage modes has been rarely studied. Therefore, in this study, we hypothesized that the deep ploughing with compaction at sowing can improve the seedling quality with better evenness and strength in winter wheat. To such end, a two-year field experiment with different tillage modes was conducted to, investigate the seedling population, leaf chlorophyll content and leaf area of crop plant, the root number and root dry weight of seedlings, yield and yield components of winter wheat grown under lime concretion black soils.

## 2. Materials and methods

### 2.1. Experimental site

**Fig 1:**
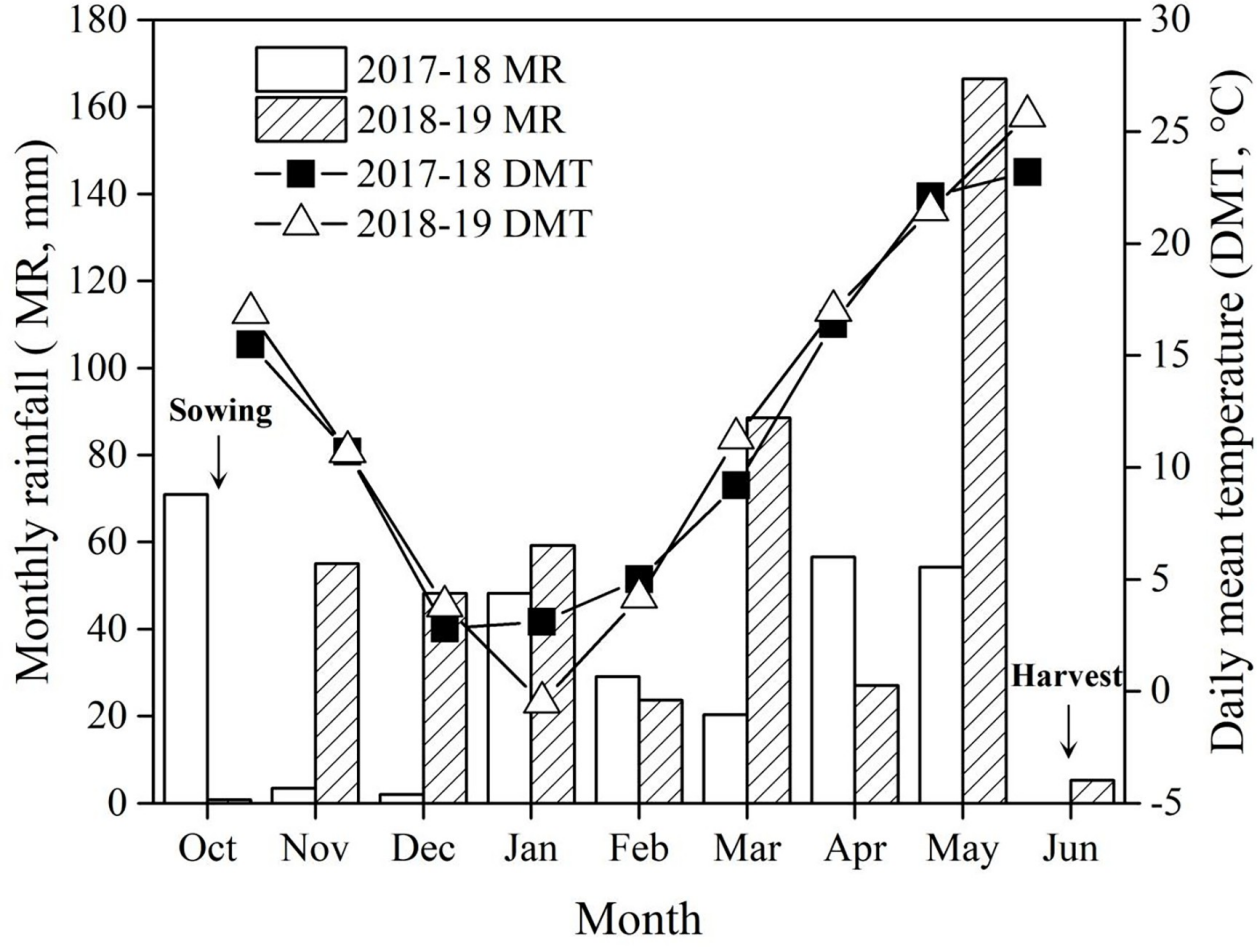
Monthly rainfall and temperature during the two growing seasons of wheat from October to June in 2017–18 and 2018–19.

A two-year field experiment was conducted at the experimental station of Mengcheng Agricultural Scientific Institute (33°9′44″ N, 116°32′56″ E), located in the Huaibei Plain region, Anhui Province, China in 2017–18 and 2018–19 winter wheat growing seasons. It has a typical semi-humid, monsoon-prone climate with an average annual precipitation of 821.5 mm. During winter wheat growing seasons, the total precipitation was 284.7 mm in 2017–18 and 474.4 mm in 2018–19, respectively (Fig 1). The soil type is a typical lime concretion black soil, with the clay content accounting for over 30%. In this two-year trial, winter wheat was grown after summer maize was harvested. Prior to the experimentation, soil physical and chemical properties were tested in two consecutive years. The soil pH was 7.5 in 2017–18 and 7.2 in 2018–19, and soil organic matter content, available N, available K and available P were 122.1 mg kg^−1^, 96.3 mg kg^−1^, 99.87 mg kg^−1^ and 15.35 mg kg^−1^ in 2017–18 and 215.6 mg kg^−1^, 84.4 mg kg^−1^, 78.2 mg kg^−1^ and 23.3 mg kg^−1^ in 2018–19 at the top 30 cm soil layer, respectively.

### 2.2. Experimental design

A randomized complete block design was used to arrange the treatments of different tillage methods, each treatment with three replications. The area of each individual experimental plot was 34.0 m^2^ (5.0 m×6.8 m). Under the maize straw retention, four tillage methods were applied prior to wheat growing seasons, namely, the traditional rotary tillage (RT), the traditional rotary tillage + compaction after sowing (RCT), deep ploughing + rotary tillage (PT), deep ploughing + rotary tillage + compaction after sowing (PCT). The compacting stone with a weight of 150 kg, was used to crush the soil. And the location of each experimental plot in the two years were kept consistent and the wheat straw was evenly returned to soil surface prior to following maize season. The winter wheat cultivar i.e. Yannong 19, as one of the most widely planted varieties in Huaibei Plain, was used in the experiment. Prior to sowing, 135.0 kg N ha^−1^, 75 P_2_O_5_ kg ha^−1^, and 75 kg K_2_O ha^−1^ were applied as the base fertilizer. Total nitrogen was 225 kg ha^−1^, with 135 kg ha^−1^ as the base fertilizer and 90 kg ha^−1^ as the top dressing at the jointing stage in winter wheat. Wheat seeds were sown on October 28, 2017 and October 16, 2018, with 25 cm row spacing, and harvested on June 5, 2018 and June 10, 2019.

### 2.3. Weather data

In this experiment, precipitation varied greatly with the growing seasons, which reflects the typical rainfall characteristic at sowing or seedling growth period of winter wheat in Huaibei Plain, and there were some fluctuations in annual air temperature (Fig 1). As shown in Fig 1, precipitation during the wheat growing season in 2018–19 was higher than that in 2017–18. However, the rainfall in October 2017 was 70.9 mm, and was only 0.8 mm in October 2018. In 2017–18 growing season, the soil moisture was sufficient for wheat sowing due to the enough precipitation before sowing, while there was little precipitation after sowing as the total rainfall was 5.4 mm from November to December. In 2017–18 growing season, there was little precipitation at sowing, in order to ensure wheat seed germination, we used irrigation of 30 mm after sowing, and the rainfall was 103.3 mm during November to December. Drought stress often happens at sowing stage or seedling stage of winter wheat in Huaibei Plain.

### 2.4. Soil moisture content

Soil moisture content (SMC) was measured prior to sowing and at two weeks after sowing (seedling stage), and the samples were collected from 0−20 cm soil layer with a soil core sampler in each plot. The soil gravimetric water content (g water g^−1^ dry soil) was assayed by oven-drying samples at 105°C for 48 h to a constant weight [29].

### 2.5. Seedling characteristics

#### 2.5.1. Seedling emergence and quality

The emergence of seedlings was investigated under different cultivation treatments daily, starting from 7^th^ day after sowing (DAS), and ended at the fifteenth DAS with 1 m^2^ of area, composed of 1 m length and 4 lines adjacent wheat plants, was randomly selected from each experimental plot and then marked. The number of seedlings at each treatment in the marked investigation area was counted daily in this study.

The number of wheat seedlings (NS), number of the germinated seeds in soil (NGS), number of ungerminated seeds but in soil (NUG), number of ungerminated seeds exposed on the soil surface (NUGS), number of dead wheat seedlings (ND), number of wheat seedlings missing (NSM, missing length≥10cm) and average distance of wheat seedling deficiency (AD) were investigated at three weeks after sowing, and the survey area of each plot is 1 m^2^.

#### 2.5.2. Seedling population and tiller number

Seedling population and tiller number were investigated at 20 DAS, and the seedling population and tillers of plant were investigated at 50 DAS and 80 DAS in this study. The sampled area of each experimental plot is 0.5 m^2^.

#### 2.5.3. Leaf area and chlorophyll content of seedlings

Five plants per plot were destructively sampled to determine leaf length (L), maximum width (W) at 20 DAS, 50 DAS and 80 DAS. LAI was calculated as 0.70×L×W [30].

The SPAD-502 chlorophyll meter was used to conduct nondestructive measurements for leaf relative chlorophyll content [31]. SPAD values correlate well with the actual chlorophyll concentration in leaf tissue [32]. The leaf chlorophyll content of five plants in each experimental plot was determined by a SPAD-502 Plus chlorophyll meter (Konica-Minolta Sensing, Osaka, Japan) when the leaf was fully expanded, and it was decided by the mean SPAD value of the upper, middle and lower parts of the leaves.

### 2.6. Shoot and root morphology and dry weight accumulation

Wheat plants in adjacent two lines with one meter (0.5 m^2^) were sampled in each plot at 80 DAS (over-wintering period). Root extraction was performed using the soil core method according to Böhm (1979) [33], and samples were washed, and sieved, then scanned and analyzed by WinRHIZO software (Version4.0b, Regent Instruments Inc., Canada). Five representative plants were chosen to determine plant height, above-ground dry matter weight of plant, root number, root length and root dry weight of plant, and the sampling depth is 20 cm. The dry matter of shoot and root was measured by oven-drying samples at 75°C for 48 h to a constant weight.

### 2.7. Grain yield and yield components

To determine grain yield, spikes were counted in 2 m^2^ prior to harvest. The grain number per spike was determined by counting the grains in each spike from 50 randomly selected plants in each plot prior to harvesting. Wheat plants from a 4 m^2^ area in each plot were harvested at maturity and threshed to determine grain yield. Actual grain yield was reported on a 13% moisture basis. The 1000-grain weight (TGW) was calculated by weighing 1000 grains from each sample and averaging three replicates.

### 2.8. Data analysis

The effects of different tillage methods on grain yield, yield components, tillage depth, soil moisture content, seedling quality, seedling population size, plant dry and root etc. Data were analyzed by analysis of variance using the GLM procedure in SPSS ver. 19.0 (SPSS, IBM, USA). Differences were judged by the least significant differences test using a 0.05 level of significance. All figures were created using OriginPro 2016 Version 9.3 (OriginLab Corp., Northampton, MA, USA).

## 3. Results

### 3.1. Tillage depth

Prior to winter wheat sowing, the tillage depth of different treatments was presented, based on 5 points investigated in each plot (Table 1). From the investigated results, year type had no significant effect on the tillage depth. The tillage method had a significant effect on the tillage depth, compared with rotary tillage, deep ploughing tillage significantly increased tillage depth. The average tillage depth was only 11.5 cm in rotary tillage, and it nearly doubled the depth in ploughing mode. The tillage depth of compaction after sowing was significantly decreased by 22.0% compared to no compaction (NC).

**Table 1:** Effect of different tillage methods on tillage depth and soil moisture content (SMC) at seedling stage in winter wheat.

Year	Treatment	Tillage depth (cm)	SMC before sowing (%)	SMC at seedling stage (%)
2017–18				
	RT	13.4 c	22.0 a	14.0 c
	RCT	9.53 d	22.0 a	16.8 b
	PT	23.6 a	22.1 a	17.2 b
	PCT	19.3 b	22.0 a	18.6 a
2018–19				
	RT	13.5 c	17.4 a	11.5 d
	RCT	9.57 d	17.4 a	14.7 b
	PT	23.6 a	17.6 a	14.0 c
	PCT	19.4 b	17.5 a	17.1 a
Analysis of variance				
F values	Y	NS	**	**
	T	**	NS	**
	C	NS	NS	**
	Y*T	NS	NS	NS
	Y*C	NS	NS	NS
	T*C	**	NS	NS
	Y*T*C	**	NS	NS

RT, the traditional rotary method; RCT, rotary with compaction after sowing; PT, rotary after deep ploughing; PCT, the rotary after deep ploughing with compaction after sowing; R, the rotary tillage method; P, the ploughing tillage method; NC, no compaction after sowing; C, compaction after sowing; The values followed by the same letter within a column in each year are not significantly difference at *P*<0.05, as determined by the LSD test. NS, the difference is not significant at *P*>0.05. **, the difference is extremely significant at *P*≤0. 01. This note also applies to the subsequent table.

### 3.2. Soil moisture content

As shown in Table 1, soil moisture content (SMC) prior to sowing was 22.0% in 2017–18 and 17.5% in 2018–19, respectively. The SMC at sowing and seedling stage in 2017–18 was significantly higher than in 2018–19 growing season due to greater rainfall difference in first year (Fig 1). There was little difference in SMC among different treatments prior to sowing. However, tillage methods had a significant effect on SMC at the seedling stage. The ploughing or compaction after sowing treatment significantly increased the SMC at the seedling stage compared to RT, of which, PCT obtained the highest SMC. Compared to RT, deep ploughing tillage significantly improved SMC at seedling stage, and the compaction was beneficial to maintain the SMC at seedling stage compared to without compaction treatments. SMC averaging 12.8% in RT in two years at seedling stage, was increased by 23.9% in RC, 22.3% in PT and 40.8% in PCT, respectively. Overall, deep ploughing tillage or compaction after sowing could significantly improve SMC at seedling stage, and PCT did the best.

### 3.3. Seedling emergence characteristics

As shown in Table 2, apart from the number of wheat seedlings missing (NSM) and the number of un-germinated seeds in soil (NUG), there was no significant difference in number of winter wheat seedlings (NS), number of the germinated seeds but in soil (NGS), seed number of un-germinated seeds exposed on the soil surface (NUGS), number of dead wheat seedlings (ND) and average distance of wheat seedling deficiency (AD) after sowing three weeks between 2017–18 and 2018–19 growing season. In 2018–19, NUG and NSM were significantly higher than that in previous year. The number of winter wheat seedlings in RT was the lowest among all the treatments in both years, and average number was 152.3 plant m^-2^; NS in RCT and PT was significantly greater compared to RT, and there was no great difference between RCT and PT, and they were all significantly lowered than that of PCT. However, compared to RT, NS was increased by 31.1%, 33.0% and 60.0% in RC, PT and PCT, respectively. Across the two years investigated, NGS in RCT and PT was significantly reduced than those in RT, and there was no significant difference between RCT and PT, but they were greatly higher than PCT. No significant difference was observed in NUG and NUGS among different treatments in this trial. There was little gap in ND and AD among RT, RCT and PT, but they significantly higher than PCT. It was significantly increased in NSM of RT than RCT, RCT significantly higher than PT, and PCT was the lowest. From the results of analysis of variance, compared with rotary tillage, deep ploughing tillage significantly increased NS, but decreased the NGS, NUGS, ND, NSM and AD. The NS in compaction after sowing was significantly increased than those of no compaction (NC), and the NGS, ND, NSM and AD were significantly decreased in NC. In brief, the deep ploughing before sowing combined with compaction after sowing (PTC) achieved the highest NS at the seedling stage at three weeks after sowing, mainly due to the least NGS, ND, NSM and AD.

**Table 2:** Effects of different tillage methods on the seedling quality in winter wheat.

Year	Treatment	NS (m2)	NGS (m2)	NUG (m2)	NUGS (m2)	ND (m2)	NSM (m2)	AD (cm)
2017–18								
	RT	159.3 c	127.3 a	38.0 a	26.7 a	25.3 a	22.7 a	12.4 a
	RCT	208.0 b	82.0 b	37.3 a	26.7 a	21.3 a	12.0 b	12.1 a
	PT	205.3 b	86.7 b	38.0 a	22.7 a	22.0 a	4.0 c	10.7 a
	PCT	246.7 a	50.0 c	35.3 b	22.7 a	18.6 a	0.0 d	0.0 b
2018–19								
	RT	145.3 c	138.0 a	43.3 a	27.3 a	30.7 a	30.7 a	13.2 a
	RCT	196.7 b	91.3 b	40.7 ab	26.7 a	27.3 ab	20.0 b	12.2 ab
	PT	199.3 b	93.3 b	40.7 ab	23.3 a	25.3 ab	10.7 c	11.4 b
	PCT	240.0 a	58.7 c	36.7 b	24.0 a	22.7 b	0.0 d	0.0 c
Analysis of variance								
F values	Y	NS	NS	*	NS	**	NS	NS
	T	**	**	NS	**	*	**	**
	C	**	**	NS	NS	NS	**	**
	Y*T	NS	NS	NS	NS	NS	NS	NS
	Y*C	NS	NS	NS	NS	NS	NS	NS
	T*C	NS	NS	NS	NS	NS	NS	**
	Y*T*C	NS	NS	NS	NS	NS	*	**

NS, the number of winter wheat seedlings; NGS, number of germinated seeds but in soil; NUG, number of un-germinated seeds in soil; NUGS, number of un-germinated seeds exposed on the soil surface; ND, number of dead wheat seedlings; NSM, number of wheat seedlings missing (≥10cm); AD, average distance of wheat seedling deficiency.

**Fig 2:**
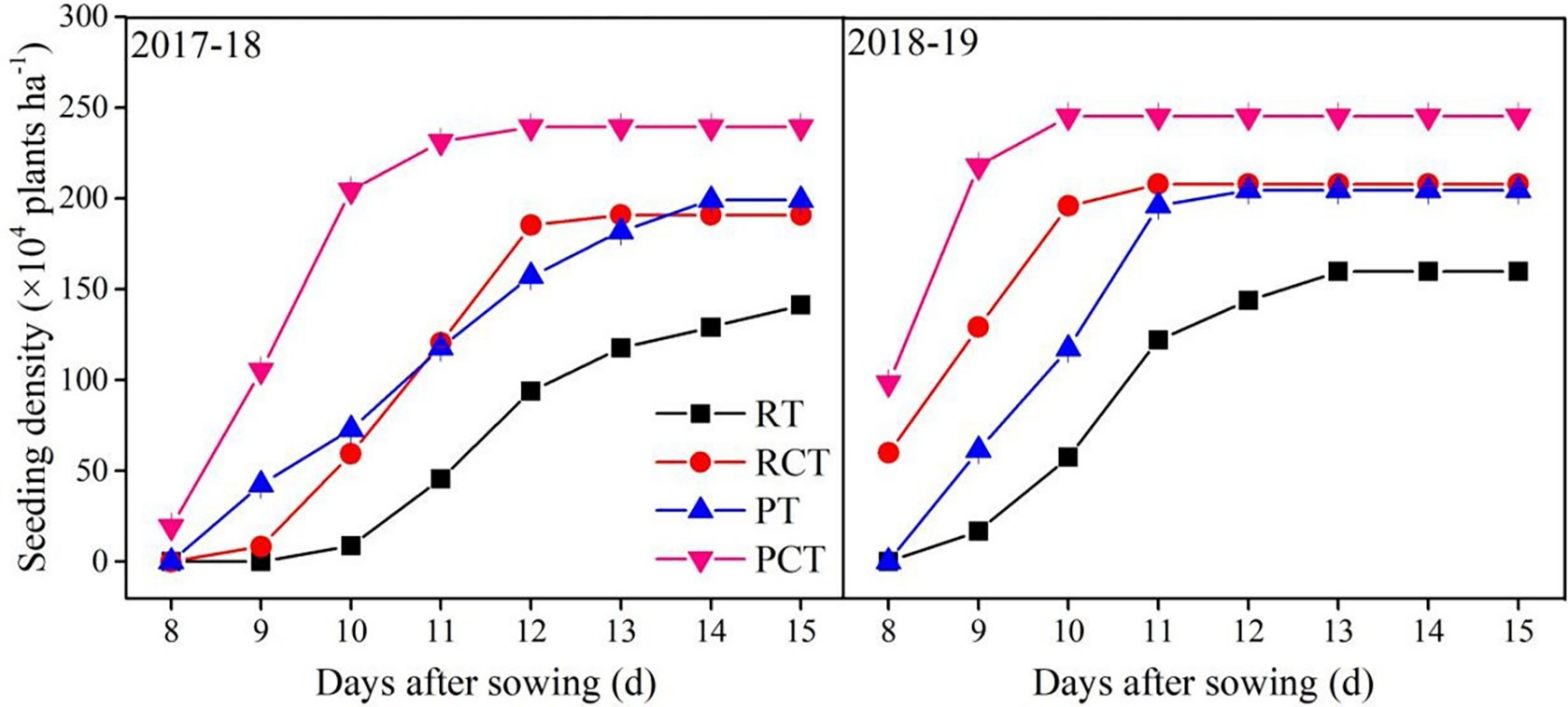
Effect of different tillage methods on seed emergence dynamics after sowing in 2017–18 and 2018–19 growing seasons. Note: RT, the traditional rotary method; RCT, rotary with compaction after sowing; PT, rotary after deep ploughing; PCT, the rotary after deep ploughing with compaction after sowing. Vertical bars represent standard errors.

Different tillage methods had a significant effect on the dynamic characteristics of seedling emergence in winter wheat (Fig 2). From the two years’ investigation, RCT, PT and PCT significantly improved the seed germination compared to RT, particularly in PCT. However, compared to no compaction (RT and PT), compaction after sowing (RCT and PCT) significantly improved seed germination. The seedling appeared at 11^th^ and 10^th^ day after sowing in 2017–18 and 2018–19 under compaction treatment (CT), while the time was delayed more than 3 days for seedlings under RT. The seedling density at 15^th^ day after sowing in PCT was the highest among all the treatments of the two years’ study, and there was no significant difference between RCT and PT, RT was the lowest. Overall, deep ploughing with compaction after sowing was more beneficial in accelerating the emergence of wheat seed and form a high seedling density of emergence stage of wheat.

The seedling population increased as the time went by after sowing (Table 3). In this study, the year type had a significant effect on the population size at 20 DAS, 50 DAS, but there was no significant difference at 80 DAS. However, the number of tillers in 2018–19 was more than the previous year. Across the two years investigated, compared with RT, the seed germination were significantly improved in RCT, PT and PCT, particularly in PCT. And three days sooner of all seedlings to appear in RCT than RT, one-day sooner in PT, and three days sooner in PCT. Apart from the population size had no significant difference between RCT and PT at 20 DAS in 2018–19, the population size in RT was significantly lower than RCT, and RCT was lower than PT, PCT was greatly higher than PT. However, there was little difference in the number of tillers at 20 DAS and 50 DAS among different treatments. At 80 DAS, the tillers of the RT and PT was significantly higher than those of RCT and PCT. From the results of analysis of variance, the population size in deep ploughing treatment (P) at 20 DAS, 50 DAS and 80 DAS were significantly increased compared to rotary treatment (R), and there was little difference in tillers per plant in different tillage modes. Compared with no compaction after sowing (NC), compaction treatment (C) significantly increased the population size, while compaction after sowing significantly decreased the number of tillers than NC. At over-wintering stage (81 DAS), the two years’ average population of wheat seedlings of PCT was 950.3 × 10^4^ ha^-1^, which increased by 57.2%, 21.6% and 20.2% than RT, RC and PT, respectively. Overall, PCT treatment was more conducive to the formation of large population at seedling over-wintering stage of wheat.

**Table 3:** Effects of different tillage methods on the population size and tillering rate of winter wheat in seedling stage.

Year	Treatment	20 days after sowing		50 days after sowing		80 days after sowing	
		Population size / (×10^4^/ha)	The number of tillers/ (plant^-1^)	Population size / (×10^4^/ha)	The number of tillers/ (plant^-1^)	Population size / (×10^4^/ha)	The number of tillers/ (plant^-1^)
2017–18							
	RT	159.0 d		318.9 d	1.99 a	636.7 d	3.95 a
	RCT	210.7 b		422.1 b	2.01 a	809.9 b	3.86 b
	PT	201.3 c		406.7 c	2.01 a	791.2 c	3.92 a
	PCT	248.7 a		501.3 a	2.01 a	961.0 a	3.87 b
2018–19							
	RT	146.0 c		293.1 d	2.00 a	574.4 d	3.98 a
	RCT	193.7 b		388.5 c	2.02 a	754.0 c	3.89 c
	PT	197.7 b		399.2 b	2.02 a	789.4 b	3.99 a
	PCT	238.7 a		484.3 a	2.02 a	939.5 a	3.94 b
Analysis of variance							
F values	Y	NS		NS	*	NS	*
	T	**		**	*	**	NS
	C	**		**	NS	**	**
	Y*T	NS		NS	NS	NS	NS
	Y*C	NS		NS	NS	NS	NS
	T*C	NS		NS	NS	NS	NS
	Y*T*C	**		**	NS	**	NS

### 3.4. Characteristics of seedling at different stages

**Fig 3:**
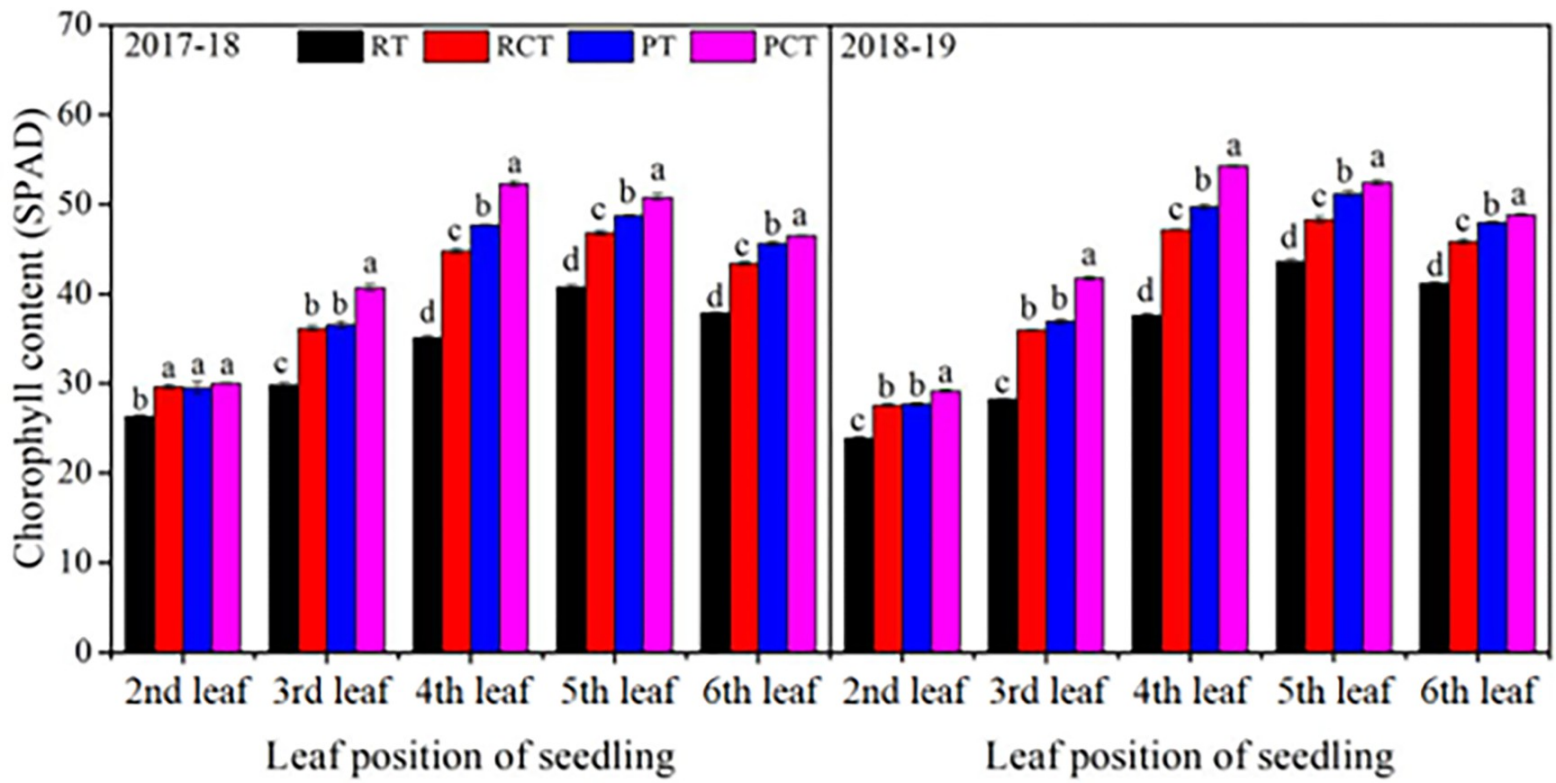
Comparisons of leaf chlorophyll content (SPAD value) under different tillage methods at seedling stage in 2017–18 and 2018–19 growing seasons. Note: RT, the traditional rotary method; RCT, rotary with compaction after sowing; PT, rotary after deep ploughing; PCT, the rotary after deep ploughing with compaction after sowing. Values followed by the same letter within a column in each year are not significantly different at *P<0*.*05*, as determined by the LSD test. Vertical bars represent standard errors.

The results of the two-year experiment showed that the chlorophyll content in leaf first increased and then had little difference with the increase of seedling leaf position in all treatments (Fig 3). In 2017–18, the chlorophyll content of 2^nd^ leaf was not significant different for RCT, PT and PCT, but they were significantly higher than RT. However, in 2018–19, the chlorophyll content of the 2^nd^ leaf in PCT was significantly higher than those of all other treatments while the content for RT was significantly lower than those in RCT and PT. The chlorophyll results for the 3^rd^ to 6^th^ leaf, the different treatments were consistent for the two years. In the 3^rd^ leaf, RT was significantly lower compared to RCT and PT, and there was no significant difference between RCT and PT, but they greatly lower than PCT. In 4^th^ to 6^th^ leaf, the chlorophyll content in RT was significantly lower compared to RCT, and RCT lower than PT, while PT lower than PCT. In brief, the highest chlorophyll content of different leaves was achieved in PCT treatment.

**Fig 4:**
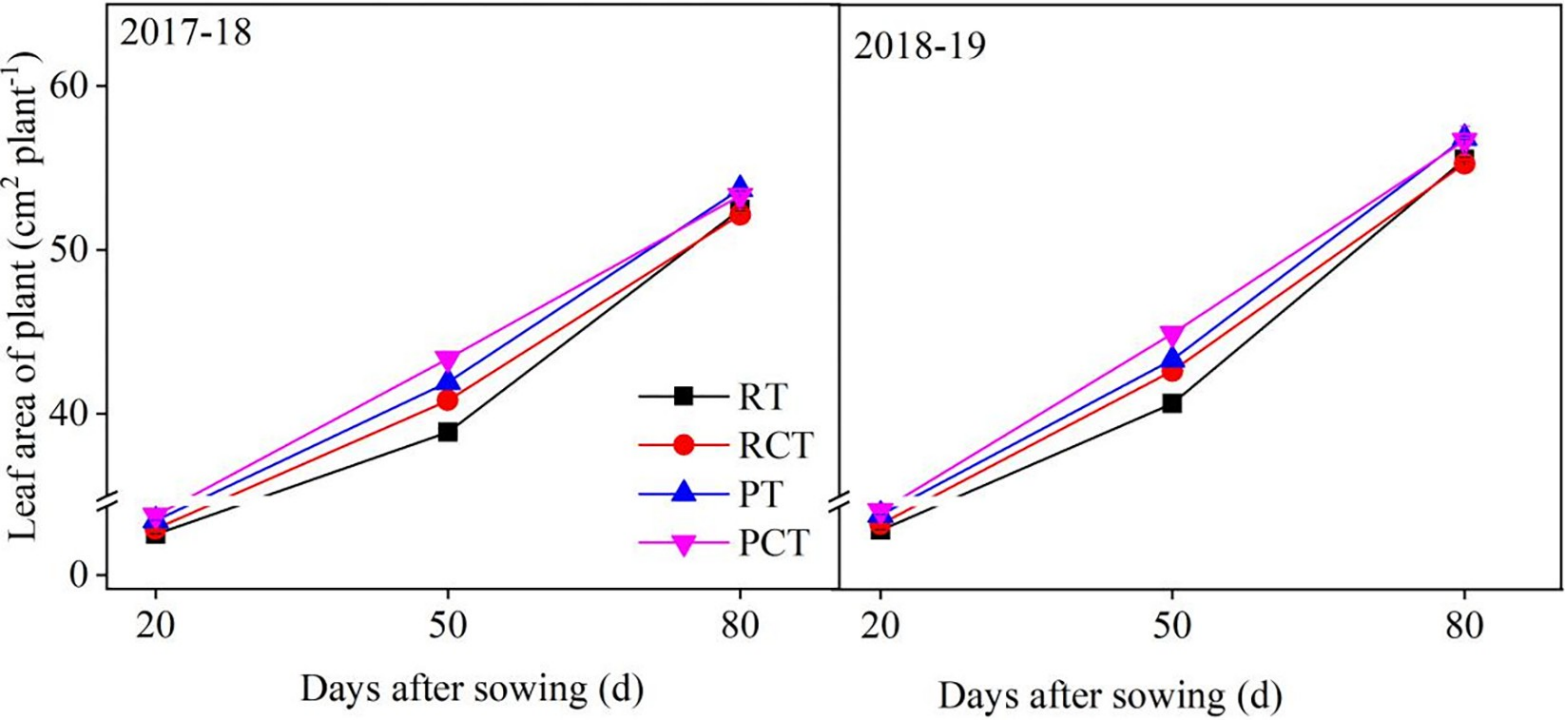
Comparisons of leaf area of seedlings under different tillage methods in 2017–18 and 2018–19 growing seasons. RT, the traditional rotary method; RCT, rotary with compaction after sowing; PT, rotary after deep ploughing; PCT, the rotary after deep ploughing with compaction after sowing. Vertical bars represent standard errors.

The leaf area of the wheat plant showed weak difference between the two growing seasons (Fig 4). However, significant difference was observed among different tillage methods. Deep ploughing method (PT or PCT) was more beneficial to accelerate the increase in plant’s leaf area compared to the rotary method (RT or RCT) at 50 DAS and 80 DAS, and compaction after sowing is also conducive to improve leaf growth and increase leaf area. In this study, the leaf area of plant in PC and PCT was significantly higher than that RT and RCT, and PCT obtained the highest leaf area of winter wheat plant at overwintering stage.

Although there were some differences in plant height, dry weight, root number, root length and dry weight under different tillage methods during the two years, the changes were essentially consistent (Table 4). The plant height was significantly lower under RT than under RCT and PT, there was little difference between RCT and PT, and they were significantly lower than PCT. Dry weight of RT was significantly lower than RCT, RCT was lower than PT and PCT, and there was little difference between PT and PCT. The root number and root length under RT and RCT had no significant difference, whereas they were significantly lower than PT and PCT, and the latter two had little difference. The root dry weight was significantly lower under RT and RCT than under PT and PCT, and it was significantly higher than PT and PCT in 2017–18, while there was little difference between PT and PCT in 2018–19. From the results of analysis of variance, plant height, dry weight, root length and root dry weight in 2018–19 was higher compared to 2017–18, but there was no significant difference in root number. However, compared with rotary method, the deep ploughing method significantly increased plant height, dry weight, root number and root dry weight after the winter dormancy stage. Compaction after sowing was instrumental in formation of higher seedling height compared to no compaction. Overall, deep ploughing with compaction after winter wheat sowing (PCT) resulted in the most vigorous seedlings in this study.

**Table 4:** Effects of different tillage methods on plant and root growth of winter wheat in over-wintering period.

Year		Treatment	Population size (×10^4^ / hm^2^)	Plant height (cm)	Dry weight (g plant^-1^)	Root number (plant^-1^)	Root length (cm)	Root dry weight (g plant^-1^)
2017–18								
		RT	1080.7 c	24.0 c	0.508 b	17.7 b	13.5 b	0.0631 c
		RCT	1450.7 b	25.1 b	0.505 c	17.6 b	13.5 b	0.0630 c
		PT	1445.3 b	25.2 b	0.520 a	25.2 a	13.6 a	0.0647 a
		PCT	1763.3 a	27.5 a	0.519 a	25.2 a	13.6 a	0.0642 b
2018–19								
		RT	1030.0 d	26.3 c	0.538 b	17.7 b	13.6 b	0.0668 b
		RCT	1376.7 c	27.4 b	0.535 c	17.7 b	13.6 b	0.0665 b
		PT	1457.3 b	27.6 b	0.550 a	27.0 a	13.8 a	0.0684 a
		PCT	1772.7 a	29.9 a	0.549 a	27.0 a	13.9 a	0.0682 a
	Analysis of variance							
F values		Y	NS	NS	*	NS	NS	NS
		T	**	**	**	**	**	**
		C	**	NS	NS	**	NS	NS
		Y*T	NS	NS	NS	NS	NS	NS
		Y*C	NS	NS	NS	NS	NS	NS
		T*C	NS	NS	NS	**	NS	*
		Y*T*C	*	**	**	**	**	**

### 3.5. Grain yield (GY) and yield components

GY, grain number per spike (GN) and thousand grain weight (TGW) were significantly affected by year type, and GY was significantly higher in 2018–19 than that in 2017–18 growing season (Table 5), due to higher GN and TGW in 2018–19. As shown in Table 5, RT yielded the lowest harvest at maturity among all the treatments, in which grain yield was only 6774.8 kg ha^-1^ and 7579.8 kg ha^-1^ in 2017–18 and 2018–19 growing season, respectively. Compared with RT, GY in RC, PT and PCT was increased by 5.53%, 9.95% and 16.7%, respectively in 2017–18, and increased by 6.21%, 10.8% and 16.0%, respectively in 2018–19. Across the two years results, compared to RT, the SN of RCT was significantly higher, while the SN of PT was higher compared to RCT, and PCT was the highest; the GN was significantly higher under RT than under RCT, while RCT was higher than those of PT and PCT, and there was no significant difference between PT and PCT. In 2017–18, the TGW in RT was significantly increased than RCT, there was little difference between RT and PT, and TGW in RCT was significantly higher than that in PCT. In 2018–19, there was no difference between RT and PT, RCT was similar to PCT, but the former was significantly higher than the later. However, compared with rotary method (R), the percentage of GY in deep ploughing method increased by 10.3%, which mainly was due to the significant increase in SN. The percentage of GY of compaction after sowing increased by 5.74% compared to no compaction, and it significantly increased SN, while decreased GN. Across the two years, PCT had the highest GY, the increase percentage in production was 16.4% compared to the traditional tillage method, and the increase in grain yield mainly owing to the raise in SN, which was up to 31.1%, while it significantly decreased the GN and TGW under PCT.

**Table 5:** Effect of different tillage methods on grain yield and yield components of winter wheat.

Year	Treatment	Grain yield (kg ha^-1^)	Spike number (×10^4^ ha^-1^)	Grain number (spike^-1^)	Thousand grain weight (g)
2017–18					
	RT	6774.8 d	562.0 c	29.5 a	40.8 a
	RCT	7149.5 c	653.4 b	28.1 b	39.0 b
	PT	7499.1 b	652.8 b	28.6 b	40.4 a
	PCT	7908.0 a	727.8 a	28.2 b	38.6 c
2018–19					
	RT	7579.8 d	557.7 c	30.6 a	44.4 a
	RCT	8050.7 c	640.4 b	29.5 b	42.6 b
	PT	8400.6 b	647.9 b	29.5 b	44.0 a
	PCT	8792.2 a	728.0 a	28.9 b	41.8 c
Analysis of variance					
F values	Y	**	NS	**	**
	T	**	**	NS	NS
	C	NS	**	**	*
	Y*T	NS	NS	NS	NS
	Y*C	NS	NS	NS	NS
	T*C	NS	NS	NS	NS
	Y*T*C	NS	NS	*	NS

## 4. Discussion

Different tillage methods caused great variations in the rate of seed germination and the quality of wheat seedling at over-wintering after the maize straw incorporation. In this present study, PCT could improve the seed emergence rate, the quality of seedling in the lime concretion black soil area mainly via the greater soil water moisture, and cause accelerated seed emergence and seedling leaf growth, the stronger roots of seedling, thus alleviating the adverse effects of drought on seed germination and growth.

### 4.1. PTC increased seeding emergence of winter wheat by ensuring suitable SMC

The soil was too loose to contact the seed closely with soil in RT due to large amount of maize residue, causing reduced soil bulk density, increased soil porosity, and accelerated soil moisture evaporate ion [9], which hindered the process of seed germination and thus reduced the emergence rate of wheat seedling [34]. The tillage method had a significant effect on the tillage depth, with deep ploughing tillage significantly increased tillage depth, which can enhance infiltration and water storage capacity [35]. The proper SMC in topsoil was conducive for seed germination, ensuring that suitable SMC during seedling stage could promote the seedling growth and development [4]. It is reported that the soil water storage increased under deep ploughing in semiarid non-irrigated farming region, resulting in a higher grain production [36]. Although the top soil in PT was also loose as in RT, the better soil tilth as resultant of rotary tillage followed by deep ploughing assured better seed-soil contact and root anchorage facilitated an increase in plant population. In addition, soil compaction can attenuate the impact of adversity stress on seedling emergence [37], but with the increase of compaction strength form low to high, the seeding emergence turns from increase to a declined [38]. In this study, the low compaction strength after sowing can increase seeding emergence rate and greater population of winter wheat. As such, seedling maximal population can be achieved in PCT, which lays a foundation for high production.

### 4.2. PCT boosted the seedling quality of winter wheat by improving root growth

The seedling quality of winter wheat is critical for its resistance to adverse environments during the subsequent growth stage. The air temperature for wheat seedling growth decreased after sowing, so the early emergence is favorable to prolong the time from seedling to over-wintering producing stronger seedlings and greater number of tillers. In our study, PCT and RCT significantly increased the seeding emergence, possibly by maintaining optimal soil moisture for seed germination and thus accelerated to reach the maximum seedling density (SD). The leaf chlorophyll content and leaf area of seedlings were significantly increased by deep ploughing, and the compaction after sowing can increased soil water content (soil water availability) at 10 and 30 cm soil depths compared to the non-compacted treatment [39] and further beneficial to root growth, so PCT treatment achieved the optimal results. In addition, the root system of the seedling begins to grow after germination, and the root system grows rapidly when entering the tillering period, which was greatly affected by soil water content [40]. What’s more, the root depth and dry weight at over-wintering was increased in PCT and PT than in RCT and RT. Thus, deep ploughing tillage prior to sowing with compaction after sowing can improve the wheat seedling quality, enhance the number of tillers and increase the depth of root system, which can be used as the recommended farming mode in lime concretion black soil area.

### 4.3. PCT maximised the potential of grain yield in winter wheat

Seedling number, tillering number and strong seedlings had great relationship to SN, GN and TGW of winter wheat [18, 33]. Compared with the conventional rotary tillage, crop yield was significantly increased by deep ploughing tillage under straw returning [41, 42]. In our study, GY in PCT was increased by 16.4% in the two years, mainly due to increased SN. However, we think that, in the region of lime concretion black soil in Huaibei Plain, the main reason for the significant decrease of TGW and GN was due to the significant increase of SN, which resulted in aggravating the competition among individuals of plants, and the smaller population under RT was beneficial for the individual to use more light, temperature, water and fertilizer resources (Table 5).

The different tillage techniques have great influence on the seedling emergence and growth of wheat [13]. In this study, the rapid emergence of wheat seedling after sowing and the formation of large and strong seedlings during the over-wintering period can be achieved by deep ploughing and compaction after sowing, so that the spike number of wheat can be significantly increased at maturity. Therefore, the problem of germination, uneven emergence and weak seedlings of wheat using rotary tillage under maize straw incorporation in Huaibei Plain was solved by PCT. In addition, we found that the seedling root growth was improved by PCT in the topsoil at the over-wintering (Table 4). In fact, the wheat production largely depends on rainfall in Huaibei Plain, and the farmer seldom uses irrigation after over-wintering, therefore, the root temporal and spatial distribution and its activity in this area play a critical role in the later growth for ensuring wheat production.

## 5. Conclusions

RCT, PT, PCT treatments could improve seed emergence rate and seedling quality in winter wheat. Of which, PCT achieved the fastest emergence and highest germination rate after sowing, which produces strongest seedlings whilst over-wintering. In addition, the uniformity of seedling emergence and the population of seedlings were increased in deep ploughing or compaction after sowing treatment, and PCT was the optimized treatment in this study. In the end, maximum grain yield over the two years has been achieved in PCT. The findings indicated that PCT could improve the rate of wheat seed emergence, and obtain stronger individuals, which lays the foundation for stable and high yield under the condition of straw incorporation for lime concretion black soil in Huaibei Plain, China or a similar soil type.

## Supporting information

S1 File This excel file includes tillage depth and soil moisture content (SMC) at seedling stage in winter wheat. ANOVA for tillage depth and soil moisture content (SMC) among different tillage methods at seedling stage in winter wheat is based on this dataset.
(XLSX)

S2 File This excel file includes seeding quality at seedling stage. ANOVA for the seedling quantity among different tillage methods in winter wheat is based on this dataset.
(XLSX)

S3 File This excel file includes leaf area of seedlings under different tillage methods. Comparisons of leaf area of seedlings under different tillage methods in 2017–18 and 2018–19 growing seasons are based on this dataset.
(XLSX)

S4 File This excel file includes population size at the seedling stage. ANOVA for the population size under different tillage treatments at the seedling stage in winter wheat is based on this dataset.
(XLSX)

S5 File This excel file includes leaf chlorophyll content (SPAD value) under different tillage methods. Comparisons of leaf chlorophyll content (SPAD value) under different tillage methods at seedling stage in 2017–18 and 2018–19 growing seasons are based on this dataset.
(XLSX)

S6 File This excel file includes leaf area of seedlings under different tillage methods. Comparisons of leaf area of seedlings under different tillage methods in 2017–18 and 2018–19 growing seasons are based on this dataset.
(XLSX)

S7 File This excel file includes shoot and root growth at over-wintering stage. ANOVA for shoot and root growth among different tillage treatments at over-wintering stage in winter wheat is based on this dataset.
(XLSX)

S8 File This excel file includes grain yield and yield components. ANOVA for grain yield and yield components among different tillage treatments in winter wheat is based on this dataset.
(XLSX)
